# The Human Microbiome as a Focus of Antibiotic Discovery: *Neisseria mucosa* Displays Activity Against *Neisseria gonorrhoeae*

**DOI:** 10.3389/fmicb.2020.577762

**Published:** 2020-12-03

**Authors:** Ellen L. Aho, Jenie M. Ogle, Anna M. Finck

**Affiliations:** Department of Biology, Concordia College, Moorhead, MN, United States

**Keywords:** human microbiome, antibiotic discovery, undergraduate research, antibiotic resistance, nonpathogenic *Neisseria* species, *Neisseria mucosa*, gonorrhea, secondary metabolites

## Abstract

*Neisseria gonorrhoeae* infections are a serious global health problem. This organism has developed disturbing levels of antibiotic resistance, resulting in the need for new approaches to prevent and treat gonorrhea. The genus *Neisseria* also includes several members of the human microbiome that live in close association with an array of microbial partners in a variety of niches. We designed an undergraduate antibiotic discovery project to examine a panel of nonpathogenic *Neisseria* species for their ability to produce antimicrobial secondary metabolites. Five strains belonging to the *N. mucosa* species group displayed activity against other *Neisseria* in delayed antagonism assays; three of these were active against *N. gonorrhoeae*. The antimicrobial compound secreted by *N. mucosa* NRL 9300 remained active in the presence of catalase, trypsin, and HEPES buffer, and effectively inhibited a DNA uptake mutant of *N. gonorrhoeae*. Antimicrobial activity was also retained in an ethyl acetate extract of plate grown *N. mucosa* NRL 9300. These data suggest *N. mucosa* produces an antimicrobial secondary metabolite that is distinct from previously described antigonococcal agents. This work also serves as a demonstration project that could easily be adapted to studying other members of the human microbiome in undergraduate settings. We offer the perspective that both introductory and more advanced course-based and apprentice-style antibiotic discovery projects focused on the microbiome have the potential to enrich undergraduate curricula and we describe transferrable techniques and strategies to facilitate project design.

## Introduction

Antimicrobial resistance (AMR) in *Neisseria gonorrhoeae* represents a major public health concern. This human-restricted Gram negative diplococcus is responsible for approximately 87 million cases of gonorrhea per year globally (Rowley et al., 2019). Sexual transmission of *N. gonorrhoeae* can result in infection of the female and male urogenital tract, rectum, and pharynx. In addition, infected women can transmit the organism during delivery, resulting in neonatal conjunctivitis. Treatment options are generally limited to dual therapy with two antibiotics (ceftriaxone plus azithromycin), and gonococcal strains exhibiting resistance to all recommended antimicrobial agents have recently been identified (Eyre et al., 2018; Whiley et al., 2018; Unemo et al., 2019). This pressing global problem has prompted the US Centers for Disease Control and Prevention (2012), World Health Organization, Department of Reproductive Health and Research (2012), and European Centre for Disease Prevention and Control (2019) to develop public health response plans addressing *N. gonorrhoeae* AMR, and new approaches for the prevention and treatment of gonococcal infections are desperately needed.

*Neisseria* species include *N. gonorrhoeae*, *N. meningitidis*, which can asymptomatically colonize the human nasopharynx and also cause deadly meningococcal meningitis, several nonpathogenic species that are components of the normal human oropharyngeal microbiome, and nonhuman isolates (Liu et al., 2015). The genus *Neisseria* is among the ten most abundant genera in the human microbiome (Human Microbiome Project Consortium, 2012), and co-colonization and horizontal gene transfer involving pathogenic and nonpathogenic *Neisseria* are well documented (e.g., Spratt et al., 1992; Maiden, 1998; Marri et al., 2010; Weyand, 2017). The nonpathogenic *Neisseria* are of particular interest because natural products produced by members of the human microbiome may represent a source of novel antimicrobial agents (Donia and Fischbach, 2015; Wilson et al., 2017; Milshteyn et al., 2018). For example, the commensal organism *Staphylococcus lugdunensis* found in the human nasal microbiome produces the secondary metabolite lugdunin, which displays antimicrobial activity against AMR *S. aureus* (Zipperer et al., 2016). Little is known about production of secondary metabolites by *Neisseria*, however, some nonpathogenic species have been shown to inhibit pathogens. *N. lactamica* (Deasy et al., 2015) and *N. cinerea* (Custodio et al., 2020) inhibit the colonization of *N. meningitidis* in human infection and epithelial cell culture models, respectively, and DNA released by *N. elongata* and multiple other nonpathogenic *Neisseria* species kills *N. gonorrhoeae* (Kim et al., 2019). In the project described here, we initiated a search for neisserial antimicrobial secondary metabolites by analyzing a panel of 36 nonpathogenic *Neisseria* strains for activity against other *Neisseria*, including *N. gonorrhoeae.* We identified five active isolates, all from the *N. mucosa* species group, and used inhibitor studies and chemical extraction to characterize the activity displayed by *N. mucosa* strain 9300.

This work was accomplished in a liberal arts college setting utilizing both traditional apprentice-style and course-based undergraduate research approaches that included both introductory and more advanced students. Projects focusing on AMR and antibiotic discovery have proven to be particularly effective at involving undergraduate students in meaningful research, connecting them to important social issues, and enlivening their study of various aspects of microbiology (e.g., Hoskisson et al., 2015; Smyth, 2017; Dube, 2018; Genné-Bacon and Bascom-Slack, 2018; Hernandez et al., 2018). Many prior projects focused on searching for antibiotic producing bacteria in environmental samples. For example, Tiny Earth^1^ represents a highly successful initiative in which students analyze soil for the presence of bacteria that display inhibitory activity against a panel of target strains, or “safe relatives,” closely related to a set of pathogens commonly responsible for nosocomial infections displaying AMR (Hernandez et al., 2018). Here, we offer the perspective the human microbiome represents another ecosystem that is ripe for undergraduate antibiotic discovery projects. We describe tools for screening nonpathogenic members of the microbiome for antimicrobial activity and offer a multiphasic project design for engaging different populations of undergraduate students in traditional and course-based research as a project evolves over time. The strategies, methods, and results described below demonstrate the scientific promise of this approach and offer an undergraduate research model that might be adapted and expanded by others interested in microbiome-based natural products discovery.

## Materials and Methods

### Bacterial Isolates, Growth Conditions, and Species Confirmation by *rplF* Sequence Analysis

Table 1 lists the nonpathogenic *Neisseria* strains used in this study. *Neisseria* were grown on GCB agar (Difco Laboratories) containing Kellogg’s supplements I and II (Kellogg et al., 1963) and incubated at 37°C with 5% CO_2_. Isolate identity for NRL strains was confirmed by PCR amplification and sequencing of a 413 bp fragment of the *rplF* gene followed by analysis using the PubMLST *Neisseria* database as previously described (Bennett et al., 2014; Jolley et al., 2018). The *rplF* sequences generated in this study were deposited in GenBank under the accession numbers given in Table 1. Strains were assigned to species groups as proposed by Bennett et al. (2012). The following well-established laboratory strains of *N. gonorrhoeae* were used as targets to test for antibiotic activity: ATCC 19424, ATCC 43070, FA19, F62, MS11, FA1090, and FA6140.

**TABLE 1:** Inhibitory activity of nonpathogenic *Neisseria* in delayed antagonism assays.

	Target Strains											
	
	*N. lactamica*		*N. flavescens*		*N. gonorrhoeae*		Additional *N. gonorrhoeae* strains^4^					
				
	ATCC 23970		N47		ATCC43070		FA1090	FA19	F62	19424	MS11	FA6140
				
Nonpathogenic *Neisseria* species^1,2,3^	CS	AO	CS	AO	CS	AO	AO	AO	AO	AO	AO	AO
***N. mucosa* group**												
*N. mucosa* NRL 9300 [MW166311]	+	8	+	15	+	11	10	10	9	10	11	10
*N. mucosa* NRL 9297 [MW166312]	−	−	+	12	+	10						
*N. mucosa* ATCC 25996	−	−	+	14	+	10						
*N. mucosa* ATCC 19696	−	−	−	−	−	−						
*N. sicca* NRL 30016 [MW166313]	−	−	+	9	−	−						
*N. sicca* ATCC 29256	−	−	+	11	−	−						
*N. sicca* ATCC 9913	−	−	−	−	−	−						
*N. sicca* NRL 272 [MW166314]	−	−	−	−	−	−						
***N. cinerea* group**	−		−		−							
*N. cinerea* ATCC 14685	−		−		−							
*N. cinerea* NRL 32828 [MW160215]	−		−		−							
*N. cinerea* NRL 33295 [MW160216]	−		−		−							
*N. cinerea* NRL 33683 [MW166303]	−		−		−							
*N. cinerea* NRL 33807 [MW166304]	−		−		−							
***N. lactamica* group**												
*N. lactamica* ATCC 23970	−		−		−							
*N. lactamica* NRL 36016 [MW166305]	−		−		−							
*N. lactamica* NRL 36046 [MW166306]	−		−		−							
*N. lactamica* NRL 36121 [MW166308]	−		−		−							
*N. lactamica* NRL 37168 [MW166309]	−		−		−							
*N. lactamica* NRL 37170 [MW166310]	−		−		−							
*N. lactamica* NRL37174 [MW166307]	−		−		−							
***N. subflava* group**												
*N. flava* NRL 30008 [MW166315]	−		−		−							
*N. flava* NRL 30037 [MW166316]	−		−		−							
*N. flava* NRL 9994 [MW166317]	−		−		−							
*N. flava* NRL 9298 [MW166318]	−		−		−							
*N. flavescens* NRL 30031 [MW166319]	−		−		−							
*N. perflava* NRL 9292 [MW166320]	−		−		−							
*N. subflava* ATCC 49275	−		−		−							
*N. subflava* ATCC 14221	−		−		−							
*N. subflava* NRL 9992 [MW166321]	−		−		−							
**Other isolates**												
*N. bacilliformis* ATCC BAA-1200	−		−		−							
*N. elongata* ATCC 25295	−		−		−							
*N. polysaccharea* ATCC 43768	−		−		−							
*N. animalis* ATCC 49930 (guinea pig)	−		−		−							
*N. canis* ATCC 14687 (dog)	−		−		−							
*N. denitrificans* ATCC 14686 (guinea pig)	−		−		−							
*N. weaveri* ATCC 51223 (dog bite)	−		−		−							

*^1^All species were isolated from human hosts unless otherwise indicated in parentheses.*

*^2^Species groups are as proposed by Bennett et al. (2012).*

*^3^Genbank accession numbers for *rplF* sequences generated in this study are given in brackets.*

*^4^*N. mucosa* NRL 9300 also displayed activity against each of these strains in the cross streak assay.*

*CS, cross streak assay; +, zone of inhibition present; −, zone of inhibition absent; no symbol, not done.*

*AO, agar overlay assay; values indicate average diameter of zone of inhibition (mm); −, zone of inhibition absent; no symbol, not done.*

**n* = 3 for each assay against each target.*

### Screening for Antimicrobial Activity

**FIGURE 1:**
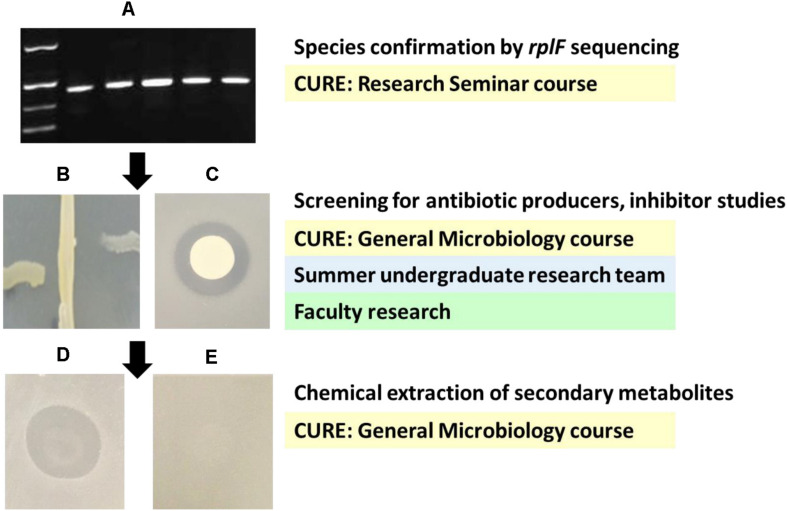
Project design and example data from each project phase. Undergraduate involvement in each phase is given in shaded boxes. **(A)** Products of *rplF* amplification. Left to right: size standard (bottom to top: 300, 400, 500, and 750 bp), PCR products from *N. mucosa* NRL 9300, *N. mucosa* NRL 9297, *N. mucosa* ATCC 25996, *N. sicca* NRL 30016, and *N. sicca* ATCC 29256. **(B)** Cross streak assay of *N. mucosa* NRL 9300 inhibiting *N. flavescens* N47 (left of center) and *N. gonorrhoeae* ATCC 43070 (right of center), *n* = 3. **(C)** Agar overlay assay of a colony of *N. mucosa* NRL 9300 inhibiting the growth of a lawn of *N. flavescens* N47, *n* = 3. **(D)** Agar overlay assay of a purified ethyl acetate extract prepared from *N. mucosa* NRL 9300 inhibiting the growth of a lawn of *N. gonorrhoeae* FA6140, *n* = 5. **(E)** Control agar overlay assay of an ethyl acetate extract prepared from uninoculated GCB agar tested against a lawn of *N. gonorrhoeae* FA6140, *n* = 5.

Delayed antagonism cross-streak assays were used to test 24-h cultures of nonpathogenic *Neisseria* for their ability to produce antimicrobial substances (Figure 1). A linear streak of each potential producer isolate was made down the center of a GCB agar plate. Plates were incubated for 48 h and target strains were then streaked at right angles to the line of original growth. Plates were examined after 24 h of additional incubation for inhibition of target strain growth in the vicinity of the central streak. A strain was considered positive for antimicrobial activity against the target if a clear zone greater than 0.5 mm was present where the target strain had been inhibited (*n* = at least three trials per strain for each target).

A confirmatory agar overlay assay was performed on all *Neisseria* strains that displayed antimicrobial activity in the initial cross streak screening. PBS (Amresco) suspensions containing 10^9^ CFU/ml of producer strains were prepared from 24 h plate cultures and 10 μl samples of the suspensions were spotted onto GCB agar. After 24 h of incubation, 10 ml of melted GCB agar containing 10^6^ CFU of a target strain was poured onto each plate and plates were reincubated. The diameter of the zone of inhibition surrounding the original producer strain was measured at 24 h (*n* = at least three trials per strain for each target). The number of CFU in the producer and target strain suspensions was confirmed by standard serial dilution and culture techniques.

### Inhibitor Studies and Chemical Extraction

Inhibitor studies were used to investigate the antimicrobial activity displayed by *N. mucos*a NRL 9300 against *N. gonorrhoeae* strains MS11 and FA6140. Agar overlay assays in which the overlay contained 5, 50, or 500 U bovine catalase (Worthington Biochemicals) per ml or 500 μg bovine pancreatic trypsin (Sigma-Aldrich) per ml were performed to inhibit H_2_O_2_ (St. Amant et al., 2002) and protein (Jyssum and Allunans, 1982), respectively. Assays in which base and top agar contained 30 mM HEPES buffer (Sigma Life Sciences) pH 7.4 were used to inhibit activity due to acidification (Knapp et al., 1975). All inhibitor assays were performed in triplicate and zones of inhibition were compared to control plates lacking inhibitors. We also tested for the possibility of DNA mediated antimicrobial activity in our agar overlay assay by testing *N. mucosa* NRL 9300 against a Δ*comP* mutant strain of *N. gonorrhoeae* MS11 that is incapable of DNA uptake. Controls for this assay included wild type strain *N. gonorrhoeae* MS11 and a complemented Δ*comP* mutant strain. All MS11 strains were generously provided by Dr. Magdalene So (Kim et al., 2019).

A crude extract containing the *N. mucosa* NRL 9300 antimicrobial compound was isolated using the technique to enrich for small, nonpolar secondary metabolites described by Hernandez et al. (2018). Briefly, a lawn of plate grown bacteria and their secreted substances were harvested by chopping the agar from a 100 mm diameter petri plate into approximately 1 cm^2^ pieces, freezing the material at −70°C, and performing an ethyl acetate extraction. The organic layer was dried, resuspended in methanol, and 30 μl samples containing 300 μg of extract, which represented approximately 20% of the yield, were spotted on an agar plate, allowed to dry, and tested against *N. gonorrhoeae* FA6140 using an agar overlay technique (*n* = 5). Control extractions were performed using uninoculated GBC agar (all chemicals were from Fisher Scientific).

## Results

### *N. mucosa* Isolates Secrete Antimicrobial Compounds

We examined a panel of 36 nonpathogenic *Neisseria* isolates representing 15 species. The collection included 32 strains isolated from the human oropharynx, including reference strains utilized in the Human Microbiome Project (HMP) (Human Microbiome Jumpstart Reference Strains Consortium, 2010), and four strains from nonhuman animals. Although all strains were obtained from established isolate collections, the NRL strains are less well studied and their species identities had not been previously confirmed by genetic analyses. We used a validated technique to analyze a 413-bp region of the neisserial *rplF* gene, which encodes the 50S ribosomal protein L6 (Bennett et al., 2014), from these strains and assigned isolates to neisserial species groups proposed by Bennett et al. (2012) (Table 1).

Delayed antagonism assays were used to test *Neisseria* species for their ability to produce antimicrobial compounds (Table 1, representative experiments shown in Figure 1). A semiquantitative cross-streak assay was used to screen all 36 isolates against three target strains: *N. lactamica* ATCC 23970, *N. flavescens* N47, and *N. gonorrhoeae* ATCC 43070. Five strains, all of which are members of the *N. mucosa* species group, displayed antimicrobial activity, although they did not exhibit identical activity profiles with respect to the target strains they inhibited (Table 1). Members of other neisserial species groups failed to show antineisserial activity under these conditions. Confirmatory agar overlay assays against the same three targets were carried out on all members of the *N. mucosa group*, yielding activity profiles identical to those seen in the initial screening experiments. Agar overlay zone of inhibition measurements suggest the *N. mucosa* producer strains may also differ from one another in their levels of antimicrobial activity, although these data should be interpreted with caution due to possible strain specific differences in growth rate and the limited quantitative power of this assay. The producer strain active against all screening targets, *N. mucosa* NRL 9300, was further tested against six additional *N. gonorrhoeae* strains, including the antibiotic resistant strain FA6140 (Veal et al., 2002), and displayed comparable activity against all gonococci in both assays (Table 1). Collectively, these data show nonpathogenic *Neisseria* species vary in their ability to secrete inhibitory compounds, and some *N. mucosa* strains are active against *N. gonorrhoeae*.

### *N. mucosa* NRL 9300 Activity Is Not Affected by Common Inhibitors and Can Be Isolated by Chemical Extraction

Inhibitor studies were used to characterize the antimicrobial activity displayed by *N. mucosa* NRL 9300. Agar overlay assays against the target strains *N. gonorrhoeae* MS11 and FA6140 were performed in the presence of catalase, trypsin, and HEPES buffer. *N. mucosa* NRL 9300 displayed activity identical to that seen on control plates lacking inhibitor in all cases (Supplementary Table 1), suggesting the inhibitory activity is not due to H_2_O_2_, a trypsin sensitive protein toxin, or acidification. Kim et al. (2019) showed nonpathogenic *Neisseria* can kill *N. gonorrhoeae* in coculture assays via a mechanism in which *N. gonorrhoeae* takes up commensal DNA. These authors also demonstrated gonococcal DNA uptake mutants deficient in the protein ComP are no longer killed by this mechanism. We tested *N. mucosa* NRL 9300 inhibitory activity against a Δ*comP* mutant strain of *N. gonorrhoeae* MS11 using our agar overlay assay. *N. mucosa* inhibited the mutant strain to the same degree that it inhibited wild type MS11 and a complemented Δ*comP* mutant strain (Supplementary Table 1), indicating the inhibition detected by our delayed antagonism assays is not due to a DNA uptake mediated mechanism. Finally, an ethyl acetate extraction procedure was performed on plate-grown *N. mucosa* NRL 9300. The resulting crude extract inhibits *N. gonorrhoeae* strain FA6140 in an agar overlay assay (Figure 1), suggesting the antimicrobial activity displayed by *N. mucosa* may be due to a novel secondary metabolite.

## Undergraduate Involvement and Transferrable Strategies

This project was designed to provide engaging undergraduate research experiences to as many students as possible while advancing the scientific objectives of the study. Although assessment of student learning outcomes was not a component of this work, a brief description of the project design and phasing from a pedagogical perspective may serve as a helpful model to others interested in designing human microbiome based antibiotic discovery experiences in undergraduate settings (Figure 1). The work described above involved over 70 students enrolled in three undergraduate biology classes in course-based undergraduate research experiences (CUREs) and provided a full-time summer research opportunity to a team of two students. Straightforward, relatively low-cost techniques were used throughout the project. Students involved in CUREs worked exclusively with nonpathogenic bacteria that could be handled safely in the teaching laboratory environment, while the faculty member and summer team carried out experiments utilizing *N. gonorrhoeae* under more stringent training and safety conditions. Participants in each research phase learned about the overarching goals of the neisserial antibiotic discovery project, and studied data generated by previous teams and relevant primary literature. Students in an upper-level genomics focused Research Seminar course carried out the DNA sequence based strain characterization in conjunction with learning about PubMLST and other databases, which they subsequently used to carry out additional independent bioinformatics projects. Participants in a General Microbiology course composed largely of students interested in health professions carried out initial screening of the isolate collection for inhibitory activity against the nonpathogenic target strains. This course represents students’ first exposure to working with bacteria in the laboratory. Participation in this study provided students with opportunities to learn not only the assays used in the project, but also basic microbiology techniques such as media preparation, streak plating, and maintaining pure cultures, in the context of an engaging research question. In addition, the project complemented classroom activities addressing symbiosis, the human microbiome, and antibiotic resistance. The summer research team, which was composed of a first year student, a third year student, and the faculty member, replicated these assays, developed the agar overlay confirmatory assay, included screening against *N. gonorrhoeae*, and carried out inhibitor studies. Finally, *N. mucosa* chemical extractions were incorporated into a subsequent section of General Microbiology. Students presented their findings from various stages of the project to the broader campus community as part of a yearly Celebration of Student Scholarship, and the summer team also presented at regional and national conferences. This phased model blending faculty research interests, course goals, and both introductory and upper-level of undergraduate research involvement has allowed a broad range of students with various levels of experience to engage with and contribute to this project.

Our laboratory has a long history of *Neisseria* research, but this undergraduate antibiotic discovery model could easily be adapted to the study of other members of the human microbiome. The NIH Human Microbiome Project included both metagenomic analyses of body site samples from human volunteers and sequencing of approximately 800 previously isolated reference strains representing an array of species found in the microbiome (Human Microbiome Jumpstart Reference Strains Consortium, 2010; Human Microbiome Project Consortium, 2012). The HMP reference strains are well described in a searchable catalog^2^ and many are readily available from commercial strain collections. While the choice to screen HMP reference strains and/or other known strain collections for antibiotic production limits the number of representatives of the microbiome one can test, it has the advantage of avoiding the need for human subject research approval and safeguards that might be challenging in some undergraduate settings. Additionally, the ready availability of many reference strain genome sequences opens the possibility of linking functional screening to bioinformatics projects related to antibiotic discovery.

## Discussion

Bacteria residing in a common habitat display a wide array of competitive and cooperative interactions. The human oropharynx is home to over 700 prokaryotic species, including at least 12 *Neisseria* species, which are in intimate contact with one another, and with their host, in variety of niches (Liu et al., 2015). Several recent studies utilizing metagenomic data from the HMP have demonstrated *N. mucosa* is a common colonizer of the healthy oropharynx and displays niche specificity for sites including the buccal mucosa and supragingival plaque (Eren et al., 2014; Kraal et al., 2014; Mark Welch et al., 2014, 2016; Donati et al., 2016). For example, Kraal et al. (2014) reported supragingival plaque contained *N. mucosa* in 70/70 subjects tested, although the abundance of the organism varied among subjects. The normal mutualistic roles *N. mucosa* and other *Neisseria* species play as members of the healthy human microbiome are not well understood, however, we reasoned that nonpathogenic *species* may secrete secondary metabolites active against other *Neisseria*. Even though our study focused on a relatively small panel of isolates, we identified five *N. mucosa* group strains exhibiting antimicrobial activity against tester strains of nonpathogenic *Neisseria*, three of which also displayed activity against *N. gonorrhoeae*. This activity was retained in a crude ethyl acetate extract enriched for small nonpolar secondary metabolites.

The *N. mucosa* antigonococcal activity we observed does not appear to be due to previously described inhibitory mechanisms. Other genera residing in human microbiome, including vaginal *Lactobacillus* species, also display activity against *N. gonorrhoeae in vitro*. *Lactobacillus*-mediated inhibition is mediated by H_2_O_2_, bacteriocins, organic acids, or a combination of the three (St. Amant et al., 2002; Graver and Wade, 2011; Ruíz et al., 2015; Foschi et al., 2017). Inhibitors of these agents had no effect on *N. mucosa* antigonococcal activity. We also used DNA uptake mutants of *N. gonorrhoeae* to rule out the possibility we were detecting DNA mediated gonococcal killing previously described in nonpathogenic *Neisseria* (Kim et al., 2019). These findings suggest further characterization of the compounds secreted by *N. mucosa* NRL 9300 is warranted and raise the possibility nonpathogenic *Neisseria* may employ multiple mechanisms for inhibiting other bacteria. This would not be surprising given that each unique nonpathogenic species interacts with a variety of other organisms in a diverse array of dynamic body site habitats.

Natural products have not been well studied in the genus *Neisseria* as a whole, and to the best of our knowledge this is the first report describing secondary metabolite activity in *N. mucosa.* Future directions for this project include chemical and quantitative analyses of the components of the *N. mucosa* ethyl acetate extract, validation of antigonoccal activity in animal models, and examination of neisserial genomes for potential biosynthetic gene clusters. The antibiotic discovery potential we have identified in *Neisseria* species is likely also present in many other uncharacterized members of the human microbiome that await investigation. This demonstration project has shown undergraduate teams participating in CUREs and other guided research experiences can make continued contributions to this emerging area of natural products research.

## Data Availability Statement

The raw data supporting the conclusions of this article will be made available by the authors, without undue reservation.

## Author Contributions

EA, JO, and AF designed and conducted experiments and interpreted the data. EA developed the overall project, directed course-based research, and wrote the manuscript. All authors contributed to the article and approved the submitted version.

## Conflict of Interest

The authors declare that the research was conducted in the absence of any commercial or financial relationships that could be construed as a potential conflict of interest.

## Footnotes

1 https://tinyearth.wisc.edu/

2 https://www.hmpdacc.org/hmp/catalog/
